# Spatio-temporal analysis of socio-economic characteristics for pulmonary tuberculosis in Sichuan province of China, 2006–2015

**DOI:** 10.1186/s12879-020-05150-z

**Published:** 2020-06-22

**Authors:** Lan Xia, Sui Zhu, Chuang Chen, Zheng-Yuan Rao, Yong Xia, Dan-Xia Wang, Pei-Ru Zhang, Jinge He, Ju-Ying Zhang, Jian-Lin Wu

**Affiliations:** 1 Department of Tuberculosis, Sichuan Provincial Center for Disease Control and Prevention, No.6 middle school road, Wuhou district, Chengdu, 610041 Sichuan Province China; 2 Department of Statistics, School of Basic Medical Sciences, Jinan University, No. 601, West of Huangpu Road, Guangzhou, 510632 Guangdong Province China; 3 Department of Epidemiology and Biostatistics, School of Public Health, Sichuan University, No.17 Section 3, Renmin South Road, Chengdu, 610044 Sichuan Province China

**Keywords:** Tuberculosis, Moran’s *I*, Hierarchical Bayesian spatio-temporal model, Social-economic factor

## Abstract

**Background:**

The disease burden caused by pulmonary tuberculosis (TB) in Sichuan province still persisted at a high level, and large spatial variances were presented across regional distribution disparities. The socio-economic factors were suspected to affect the population of TB notification, we aimed to describe TB case notification rate (CNR) and identify which factors influence TB epidemic are necessary for the prevention and control of the disease in Sichuan province.

**Methods:**

A retrospective cross-sectional study and an ecological spatial analysis was conducted to quantify the presence and location of spatial clusters of TB by the Moran’s *I* index and examined these patterns with socio-economic risk factors by hierarchical Bayesian spatio-temporal model.

**Results:**

A total of 630,009 pulmonary TB cases were notified from 2006 to 2015 in 181 counties of Sichuan province. The CNR decreased year by year since 2007, from 88.70 to 61.37 per 100,000 persons. The spatial heterogeneities of CNR were observed during the study periods. Global Moran’s *I* index varied from 0.23 to 0.44 with all *P*-value < 0.001. The Bayesian spatio-temporal model with parametric spatio-temporal interactions was chosen as the best model according to the minimum of Deviance Information Criterion (*DIC*)(19,379.01), and in which the quadratic form of time was taken. The proportion of age group and education year were all associated with CNR after adjusting the spatial effect, temporal effect and spatio-temporal interactions. TB CNR increased by 10.2% [95% credible interval (*CI*): 6.7–13.7%] for every 1-standard-deviation increase in proportion of age group and decreased by 23% (95% *CI*: 13.7–32.7%) for every 1-standard-deviation increase in education year.

**Conclusions:**

There were spatial clusters of TB notification rate in Sichuan province from 2006 to 2015, and heavy TB burden was mainly attributed to aging and low socioeconomic status including poor education. Thus, it is more important to pay more attention to the elderly population and improve socioeconomic status including promoting education level in Sichuan province to reduce the TB burden.

## Background

Tuberculosis (TB) is one of the top 10 causes of death worldwide. In 2018, there were about 10 million new cases of TB, and 1.2 million deaths among HIV-negative people according to the world health organization (WHO) [1]. China alone accounted for nearly 0.86 million or an estimated 9% of the total TB cases reported worldwide, ranked alongside India [1].

The WHO End TB Strategy outlines global targets to reduce TB deaths by 90% and to cut new cases by 80% between 2015 and 2030 [2]. Thus, it’s meaningful to explore the risk factors for TB notification rate. Researchers had indicated that there were two determinants for TB susceptibility: one for individual, and another for the population [3]. Individual factors, such as age, gender, alcohol drinking, smoking, diabetes [4–7], have influence on the occurrence of TB, which were frequently studied by cohort and case-control studies. The ecological risk factors for TB susceptibility at population level generally attracted interests from the government officials, which were important to figure out the public health response, and were frequently addressed by relevant studies.

The socio-economic factors mainly affected the population of TB susceptibility, such as poverty [8], overcrowding [9] and sex ratio [4]. However, there is no consensus on ecological factors. Mangtani indicated that overcrowding and the proportion of migrants correlated with the average level of notification rates of TB in 32 London boroughs between 1981 and 1991 [9]. Myers found that there is no association between overcrowding and unemployment with increased TB transmission after adjusting for other measures within California, using pediatric tuberculosis as an indicator of new transmission [10]. Some studies [4, 11] indicated that a higher proportion of females would result in increasing TB notification, while others did not find such association [12]. Thus, the ecological factors varied in different regions due to population heterogeneity. To explore the socio-economic risk factors for TB case notification rate (CNR), the spatio-temporal clusters should be taken into account. Some studies had revealed that the spatio-temporal clusters of reported TB cases in mainland China [13, 14], and also in the Sichuan province [15], which shown that TB had a heterogeneous distribution in space and time. Thus, if space and time were not accounted for, the risk factors identified could not be reliable. The spatio-temporal model was superior to the traditional methods to explore the socio-economic risk factors, it had been applied to explore the risk factors for malaria [16], bladder cancer [17], schistosomiasis [18] and hand-foot-mouth disease [19], however, few studies had used this model in TB field. Jafari-Koshki used the spatio-temporal models to evaluate the spatial distribution and trends in the risk of smear-positive *M.tb* in Iran during 2001–2012 [20]. Cao used six different Bayesian spatio-temporal statistical models to probe the climate factors for TB in 31 mainland China provinces during 2009–2013 [21]. Guo used a generalized linear mixed model to identify social-economic and meteorological factors associated with TB incidence rate in mainland China during 2005–2013 [11]. Although time and space effects were included in the model, the spatio-temporal interaction was not considered in these studies.

**Fig. 1:**
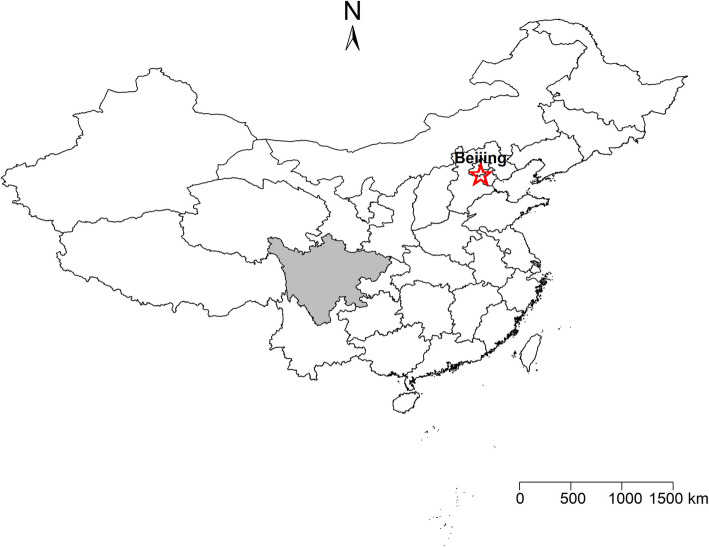
The location of Sichuan province in China. The grey part was the Sichuan province and the red star was the capital of China, Beijing

According to the fifth national TB epidemiologic survey, the highest prevalence of TB was observed in western China [22]. Sichuan province (see Fig. 1), located in the southwestern of China between latitude 26°03′N to 34°19′N and longitude 97°21′E to 108°33′E, with a population of 83.02 million and a per gross domestic product (PGDP) of 44,651 Chinese Yuan (CNY) in 2017 [23], is one of the highest TB burden provinces of TB in China. The prevalence of active pulmonary TB and smear-positive TB among 15 years old and above in Sichuan province were 598 per 100,000, 104 per 100,000, respectively. Both of them were higher than those of the national level (459 per 100,000 and 66 per 100,000, respectively) [24]. According to the 2018 statistical report of Sichuan province, the reported morbidity and mortality of TB ranked second in the class A and B notifiable infectious diseases [25].

Relatively little was known about the impact of socio-economic factors on high TB burden in Sichuan province. Thus, it’s important to explore the socio-economic risk factors for TB CNR in Sichuan province, to provide the basis for the local government to tailor its health care policy toward TB. In this study, we aim to explore the spatial cluster of CNR, and to identify the socio-economic risk factors for CNR by using the spatio-temporal model in 181 counties of Sichuan province from 2006 to 2015.

## Methods

### Study design and population

This is a retrospective cross-sectional study, and an ecological spatial analysis of TB CNR from 2006 to 2015 was conducted to quantify the presence and location of spatial clusters of notified TB cases. The population were all residents of Sichuan province, and stratified by county-level and year, which were obtained from the Sichuan province Center for Disease Control and Prevention.

### TB cases

Mandatory TB notification is requested by national TB control program in China. All pulmonary TB cases or suspected cases detected in any health facilities must be reported through the infectious disease reporting system within 24 h. Thus, all newly diagnosed active pulmonary TB cases in Sichuan province from 2006 to 2015 could be collected from the TB surveillance system. The extrapulmonary TB (2729 cases), diagnosis correction (1446 cases) and TB patients who had emigrated to other provinces (318 cases) were excluded in this study. The aggregate data at the county-level (181counties) were used to analyze the social-economic variables.

### Socio-economic variables

Five social-economic variables were considered as followings:(1) economic levels: including PGDP (1000 CNY) and annual employment rate (persons/household register); (2) demographic characteristics: including proportion of age group (≥60 years), sex ratio (male to female) and education year; (3) crowdedness: including population density (PD) (population/km^2^); (4) the degree of government attention: including local government finance expenditure for public health (1000 CNY); (5) health service: number of beds in medical institutions per 1000 persons (NBMI) and number of medical personnel per 1000 persons (NMW). The education year originated from the 2000 and 2010 census, and the constant proportion transformation model was used for interpolation and extrapolation from 2006 to 2015. The health service variable came from the health commission of Sichuan province [25], and other socio-economic variables were obtained from the Sichuan Provincial Bureau of Statistics of China [23].

### Statistical analysis

#### Descriptive analysis

The socio-economic variables of PGDP, PD, local government finance expenditure for the public, NBMI and NMW were log-transformed due to the positively skewness distribution of the variables. Then means and standard deviations *(SD)* were used to describe the socio-economic variables and Pearson linear correlation coefficients were used to explore the association among them.

#### Spatial cluster analysis

The global spatial autocorrelation test of Moran’s *I* index [19] was used to explore the spatial cluster of TB CNR from 2006 to 2015, and the Queen weights were used in this study (Additional file 1).

#### Bayesian spatio-temporal analysis

Under the spatial clustering, we used a hierarchical Bayesian spatio-temporal model to explore the socio-economic risk factors for the population TB susceptibility. The cases of TB follow a negative binomial distribution:

$$ {\displaystyle \begin{array}{cc}Y\sim NB\left(\mu, \kappa \right)\kern8.7em & \\ {}E\left({y}_{it}\right)={\mu}_{it}\kern9em & Var\left({y}_{it}\right)={\mu}_{it}+{\kappa \mu}_{it}^2\\ {}\log \left({\mu}_{it}\right)={\mu}_0+\log \left({E}_{it}\right)& \end{array}} $$

Here, *μ*_*it*_ represents the number of expected cases of pulmonary TB and *y*_*it*_ represents the reported TB cases at the *t*^th^ time point in the *i*^*th*^ region. *κ* is the over-dispersion parameter, and *μ*_0_ is the intercept, *E*(*y*_*it*_) = *μ*_*it*_ was the average number of TB, the offset variable log(*E*_*it*_) is the expected number of TB cases calculated as the product of overall TB CNR and population size of each county at a given time. And then we built five models without socio-economic covariates and used a data-driven method to select the one with the lowest Deviance Information Criterion (*DIC*) as the main model [26].

1. model I: main spatial effect.

$$ \log \left({\mu}_{it}\right)={\mu}_0+\log \left({E}_{it}\right)+{\mu}_i+{\nu}_i $$

Here *μ*_*i*_ is the spatially structured residual, and *ν*_*i*_ is unstructured residual modeled for the *i*^*th*^ region. Conditional auto-regression (CAR) is specified on *μ*_*i*_. To complete the Bayesian model, the priors on *μ*_*i*_ and *ν*_*i*_ are specified as followings.

$$ {u}_i\left|{u}_{j,j\ne i}\sim N\left(\gamma \overline{u},{\sigma}_u^2/{N}_i\right)\right. $$

The *γ* is the spatial correlation coefficient, which reflects the spatial correlation in spatial neighboring areas. $\overline{u_i}$ is the mean for the area *i.*${\sigma}_u^2/{N}_i$ is the variance for the *i*^*th*^ region, which depends on its numbers of neighbors (*N*_*i*_). When the *γ* equals to 1, this specification is called intrinsic CAR. The priors of *ν*_*i*_ follows normal distribution using exchangeablility ${v}_i\sim N\left(0,{\sigma}_{\nu}^2\right)$.

(2) model II: independently main spatial and temporal effects.

$$ \log \left({\mu}_{it}\right)={\mu}_0+\log \left({E}_{it}\right)+{\mu}_i+{\nu}_i+{\gamma}_t+{\phi}_t $$

Here *γ*_*t*_ represents the temporally structured effect, modeled dynamically using autoregressive model of order 1 (AR1):

$$ {\displaystyle \begin{array}{cc}{\gamma}_t\sim N\left.{\left(0,\tau \left(1-{\rho}^2\right)\right)}^{-1}\right)& t=1\\ {}{\gamma}_t={\rho \gamma}_{t-1}+{\varepsilon}_t\kern5em & t=2,\dots \dots \mathrm{n}\end{array}} $$

We specified the *ε*_*t*_ and temporally unstructured effect *ϕ*_*t*_ as normal distribution using exchangeablility *ε*_*t*_ ∼ *N*(0, *τ*^−1^).

(3) model III: nonparametric spatio-temporal interactions model.

$$ \log \left({\mu}_{it}\right)={\mu}_0+\log \left({E}_{it}\right)+{\mu}_i+{\nu}_i+{\gamma}_t+{\phi}_t+{\delta}_{it} $$

The space-time interaction *δ*_*it*_ represents the residual which can’t be explained by the space and time, specified as normal distribution using exchangeablility ${\phi}_{it}\sim N\left(0,{\sigma}_{it}^2\right)$.

(4) model IV: parametric spatio-temporal interactions model with linear form of time.

$$ \log \left({\mu}_{it}\right)={\mu}_0+\log \left({E}_{it}\right)+{\mu}_i+{\nu}_i+\beta {X}_t+{\delta}_{it} $$

The parametric trends for the temporal component which assume the linear form of time is taken and the *β* is the coefficient for the time.

(5) model V: parametric spatio-temporal interactions model with quadratic form of time.

$$ \log \left({\mu}_{it}\right)={\mu}_0+\log \left({E}_{it}\right)+{\mu}_i+{\nu}_i+{\beta}_1{X}_t+{\beta}_2{X}_t^2+{\delta}_{it} $$

The parametric trends for the temporal component which assume the quadratic form of time is taken, and the *β*_1_ and *β*_2_ are the coefficients for the time one and two.

In this study, the priors for the *μ*_0_ and regression coefficient *β* are typically as a normal distribution with a mean of 0 and large variability of *σ*^2^, which specified as uniform distributions *σ* ∼ *U*(0, 1000). The variances of *μ*_*i*_, *ν*_*i*_, *γ*_*t*_, *ϕ*_*t*_ and *δ*_*it*_ are specified as Jeffrey’s prior, which transform the variance into accuracy parameters *τ*, and *τ* is specified as Gamma distribution *τ* ∼ *G*(0.1, 0.1) [27]. The selected prior information was shown in Additional file 2.

Univariate analysis was used to explore the risk factors for TB CNR by building socio-economic covariate models, and the multivariable analysis was conducted to build different covariate multivariable models in combination with different socio-economics, and then the model with the lowest *DIC* was reported in this study [26]. The posteriori distributions of parameters and relative risk (*RR*) and 95% credible interval (*CI*) were fitted using Integrated Nested Laplace Approximation (INLA) methods implemented in the R package ‘INLA’ [28], and all the socio-economic variables were standardized to a z scale, which permitted the comparation among variables.

## Results

### Annual TB case notification rate

**Fig. 2:**
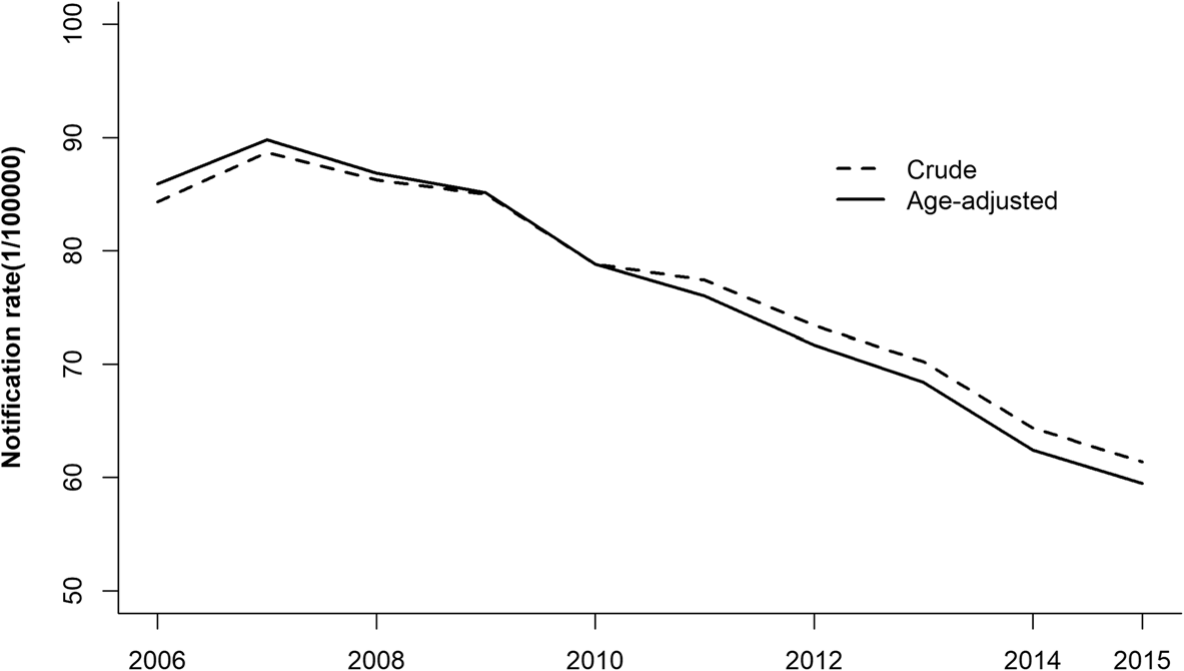
Annual TB notification rate by age standardization from 2006 to 2015 in Sichuan province. The solid line represented the notification rate by age standardization using the China population census in 2010 and the dotted line represented the crude TB notification rate

A total of 630,009 TB cases were included in this analysis. The TB CNR decreased year by year since 2007, from 88.70 to 61.37 per 100,000 persons, and the reduction in the age-standardized CNR was 30.78%, with an annual decline of 3.08% using the China population census in 2010 to standardize age (see Fig. 2).

### Characteristics of socio-economic variables

Table 1 shows the characteristics of socio-economic variables in Sichuan province from 2006 to 2015. The proportion of people over 60 years old constitutes nearly one-third of the whole population and the male-female sex ratio is more than 1. The mean value of local government finance expenditure for the public is 117 thousand CNY, and the mean value of education year is 7.81 years, which is less than the 9-year compulsory educations years. The mean value of NBMI and NMW per 1000 persons log-transformed were 1.25 and 0.99, respectively.

**Table 1:** The characteristics of the socio-economic variables in 181 counties of Sichuan province, 2006 to 2015

Variables	Mean ± ***SD***	Min	P_**25**_	P_**50**_	P_**75**_	Max
PGDP (1000 CNY)^a^	97.69 ± 6.95	80.71	92.60	97.69	102.56	117.08
The proportion of age	0.26 ± 0.06	0.12	0.22	0.26	0.29	0.40
Sex ratio (male to female)	1.04 ± 0.05	0.86	1.01	1.04	1.07	1.19
Local government finance expenditure for public health (1000 CNY)^a^	117.06 ± 8.38	93.03	111.41	117.44	123.40	140.29
PD (Population/km^2^)	5.24 ± 1.78	0.97	4.36	5.83	6.36	10.29
Education year (Year)	7.81 ± 1.59	2.17	7.31	7.93	8.57	13.04
Annual employment rate (Persons/household register)	0.64 ± 0.13	0.24	0.56	0.63	0.70	1.32
NBMI per 1000 persons^a^	1.25 ± 0.54	− 0.24	0.87	1.22	1.58	2.84
NMW per 1000 persons^a^	0.99 ± 0.57	− 0.43	0.59	0.94	1.31	2.93

*PGDP* Per gross domestic product, *PD* Population density, *NBMI* Number of beds in medical institutions, *NMW* Medical personnel per 1000 persons, *SD* standard deviation, *P*_*25*_ 25% quartile, *P*_*50*_ median, *P*_*7*_ 75% quartile

^a^The Log-transformed was used and the distributions were not normally distributed

As shown in Table 2, there are positive correlations between PGDP and education year, PD and education year, NBMI and NMW, with correlation coefficients of 0.70, 0.69 and 0.90, respectively. However, there are no correlations among the proportion of age, sex ratio, PD and annual employment rate (*P* > 0.05).

**Table 2:** The Pearson correlation coefficients of socio-economic variables in 181 counties of Sichuan province, 2006 to 2015

	The proportion of age	sex ratio (male to female)	local government finance expenditure for public health (1000 CNY)^*^	PD (Population/km^2^)	education year (Year)	annual employment rate (Persons/household register)	NBMI per 1000 persons^a^	NMW per 1000 persons^a^
PGDP (1000 CNY)^a^	0.49	−0.16	0.55	0.44	**0.70**	0.10	0.44	0.53
the proportion of age		−0.39	0.52	0.54	0.50	0.02^b^	0.10	0.10
sex ratio (male to female)		1	−0.11	−0.25	− 0.15	−0.03^b^	− 0.14	−0.18
local government finance expenditure for public health (1000 CNY)^a^			1	0.47	0.52	0.10	0.11	0.10
PD (Population/km^2^)				1	**0.69**	0.02 ^b^	0.26	0.32
education year (Year)					1	0.08	0.48	0.59
annual employment rate (Persons/household register)						1	0.15	0.14
NBMI per 1000 persons^a^							1	**0.90**

*PGDP* Per gross domestic product, *PD* Population density, *NBMI* Number of beds in medical institutions, *NMW* Medical personnel per 1000 persons

^a^The Log-transformed was used and the distributions were not normally distributed

^b^*P* > 0.05

### Spatial autocorrelation

There were high spatial autocorrelations of TB CNR from 2006 to 2015, and the Global Moran’s *I* indexes were all positive, ranged from 0.23 to 0.44, with all the *P*-value < 0.001, which indicated that the annual TB CNR was clustered among county-levels in Sichuan province (see Table 3).

**Table 3:** The spatial autocorrelations of TB CNR in 181 counties of Sichuan province, 2006 to 2015

Year	Moran’s *I*	*E(I)*	*Var(I)*	*Z* score	*P* value
2006	0.28	−0.006	0.0021	6.18	< 0.001
2007	0.23	−0.006	0.0021	5.21	< 0.001
2008	0.25	−0.006	0.0021	5.65	< 0.001
2009	0.30	−0.006	0.0016	6.68	< 0.001
2010	0.40	−0.006	0.0016	8.85	< 0.001
2011	0.40	−0.006	0.0017	8.85	< 0.001
2012	0.33	−0.006	0.0017	7.34	< 0.001
2013	0.40	−0.006	0.0021	8.85	< 0.001
2014	0.44	−0.006	0.0021	9.84	< 0.001
2015	0.40	−0.006	0.0020	9.00	< 0.001

*E(I)* Moran’s *I* expectation, *Var(I)* Moran’s *I* variance

### Model selection

**Fig. 3:**
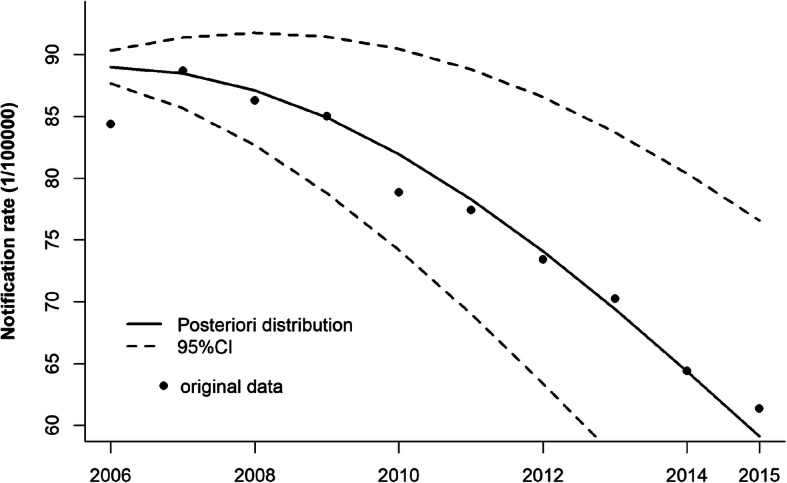
The quadratic trend between TB notification rate and time in Sichuan province from 2006 to 2015. The solid line is the fitted value using parametric spatio-temporal interactions model II: io$\log \left({\mu}_{it}\right)={\mu}_0+\log \left({E}_{it}\right)+{\mu}_i+{\nu}_i+{\beta}_1{X}_t+{\beta}_2{X}_t^2+{\delta}_{it}$ and the dotted line is the 95% credible interval for the posteriori distribution

According to the minimum of *DIC* (19,379.01), the model V with parametric trends for the temporal component which include the quadratic term of the time was chosen as the best model for spatio-temporal analysis, with prior information of uniform distribution *σ* ∼ *U*(0, 1000), and Gamma distribution *τ* ∼ *G*(0.01, 0.01) (seen Additional file 2). From Fig. 3, the scatter plot of TB CNR in Sichuan province from 2006 to 2015 showed a quadratic trend, and the posteriori distribution of TB CNR fitted well when using model V. Thus, the model V was chosen as the best model for spatio-temporal analysis in this study, and the posteriori distributions of parameters for model V without covariate were shown in Additional file 3.

### Univariate analysis

The socio-economic variables were added to the model V dependently to do the univariate analysis. There were positive association of TB CNR between the PGDP, the proportion of age, NBMI per 1000 persons and NMW per 1000 persons, however, there were negative association of CNR between the sex ratio and local government finance expenditure for public, PD, education year and annual employment rate, and the posteriori distributions of socio-economic parameters were shown in Table 4.

**Table 4:** The *DICs* and posteriori distributions of each socio-economic parameter added to the model V in 181 counties of Sichuan province, 2006 to 2015

Variables	*DIC*	Mean	*SD*	P_2.5_	P_50_	P_97.5_
PGDP (1000 CNY)^a^	19,362.542	0.062	0.03	0.004	0.062	0.121
the proportion of age	19,334.163	0.106	0.016	0.074	0.106	0.138
sex ratio (male to female)	19,366.804	−0.029	0.011	−0.05	− 0.029	− 0.008
local government finance expenditure for public health (1000 CNY)^a^	19,364.596	− 0.051	0.016	− 0.083	− 0.051	− 0.018
PD (Population/km^2^)	19,368.466	− 0.069	0.039	− 0.145	− 0.07	0.007
education year (Year)	19,310.28	−0.339	0.079	−0.509	−0.334	− 0.198
annual employment rate (Persons/household register)	19,368.987	−0.014	0.007	−0.028	−0.014	0.001
NBMI per 1000 persons^a^	19,369.852	0.006	0.008	−0.009	0.006	0.021
NMW per 1000 persons^a^	19,369.115	0.013	0.01	−0.006	0.013	0.033

*PGDP* Per gross domestic product, *PD* Population density, *NBMI* Number of beds in medical institutions, *NMW* Medical personnel per 1000 persons, *SD* standard deviation, *P*_*25*_ 25% quartile, *P*_*50*_ median, *P*_*7*_ 75% quartile

^a^The Log-transformed was used and the distributions were not normally distributed

### Multivariable analysis

Because of the high correlation among PGDP, PD and education year, NBMI and NMW (see Table 2). Thus, education year, NMW per 1000 people, the proportion of age, sex ratio, annual employment rate and local government finance expenditure for public were chosen as covariates in the multivariate analysis. In the univariate analysis, it shows that model with the education year covariate had the smallest *DIC* among those variables, the other covariates were combined with education year in the multivariable analysis exploring the best influenced risk factors.

At last, the model 1 was the final model for multivariable analysis with the lowest *DIC*, which meant education year and the proportion of age group had associations with the TB CNR spatio-temporal (see Table 5), with the posteriori distributions of parameters and *RRs* in model 1 shown in Table 6.

**Table 5:** The *DICs* of multivariable analysis with different social-economic variables for model V in 181 counties of Sichuan province, 2006 to 2015

Models	Covariates	*DIC*
**Model 1**	**education year + the proportion of age**	**19,299.517**
Model 2	education year + sex ratio (male to female)	19,315.13
Model 3	education year+ sex ratio (male to female) + the proportion of age	19,302.156
Model 4	education year + sex ratio (male to female) + local government finance expenditure for public^a^	19,301.28
Model 5	education year + sex ratio (male to female) + annual employment rate	19,300.942
Model 6	education year + sex ratio (male to female) + NMW per 1000 persons^a^	19,301.266
Model 7	education year + sex ratio (male to female) + the proportion of age + annual employment rate	19,303.596
Model 8	education year + sex ratio (male to female) + the proportion of age + local government finance expenditure for public^a^	19,303.982
Model 9	education year + sex ratio (male to female) + the proportion of age + NMW per 1000 persons^a^	19,303.825
Model 10	education year + sex ratio (male to female) + the proportion of age + NMW per 1000 persons^a^ + local government finance expenditure for public^a^ + annual employment rate	19,304.697

*NMW* Medical personnel per 1000 persons, *DIC* Deviance information criterion

^a^The Log-transformed was used and the distributions were not normally distributed

**Table 6:** The posteriori distributions of parameters for education year and the proportion of age in the model 1 in Sichuan province, 2006 to 2015

Parameter	$\hat{\beta}$	$\hat{SE}$	*RR*	95% *CI*
*μ*_0_	−6.955	0.027	–	–
*β*_1_	−0.004	0.007	0.996	0.983, 1.010
*β*_2_	−0.005	0.001	0.995	0.994, 0.996
*β*_*old*_	0.097	0.016	1.102	1.067, 1.137
*β*_*edu*_	−0.264	0.063	0.770	0.673, 0.863
*κ*	34.591	1.651	–	–
*τ*_*v*_	18.509	5.182	–	–
*τ*_*u*_	10.834	3.617	–	–
*τ*_*δ*_	310.571	35.492	–	–

*RR* Relative risk, *CI* Credible interval

*μ*_0_Constant term, *β*_1_ and *β*_2_ Coefficients for the time, *β*_*old*_ Coefficient for the proportion of age, *β*_*edu*_ Coefficient for the education year, *κ* Dispersion parameter, *τ*_*v*_ Spatially unstructured residual, *τ*_*u*_ Spatially structured residual, *τ*_*δ*_ The space-time interaction

*β*_1_ and *β*_2_ were negative for the TB CNR, which indicated that as time passed by, the TB CNR decreased in Sichuan province. TB CNR increased by 10.2% (95% *CI*: 6.7–13.7%) for every 1-standard-deviation increase in proportion of age and decreased by 23% (95% *CI*: 13.7–32.7%) for every 1-standard-deviation increase in education year after adjusted the spatial effect, temporal effect, and spatio-temporal interactions. The residues showed that the spatio-temporal model with education year and the proportion of age fitted the results well in this study (Additional file 4).

**Fig. 4:**
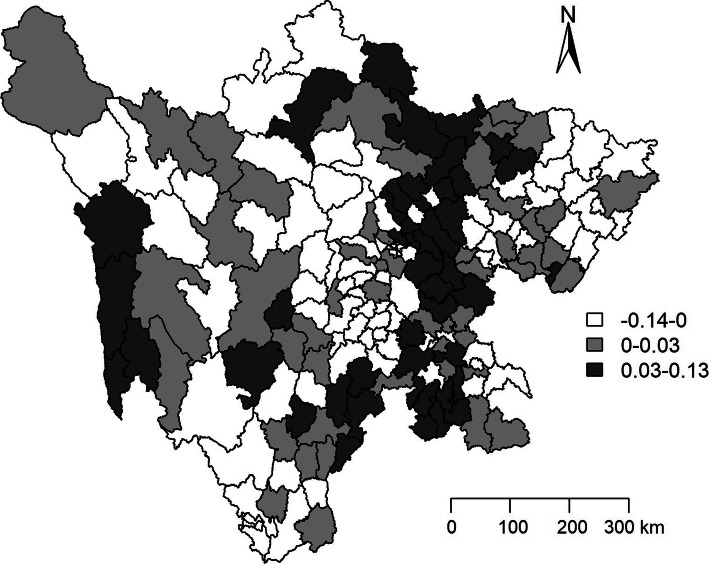
Maps of the distribution of the estimated county level posterior mean smoothed for temporal effect after adjusting the social-economic variables in Sichuan province. The deeper in the color and the higher of posteriori distribution. In comparison with the average decrease tendency of TB in Sichuan province from 2006 to 2015, the areas with dark grey had the faster decreasing of the TB notification rate

**Fig. 5:**
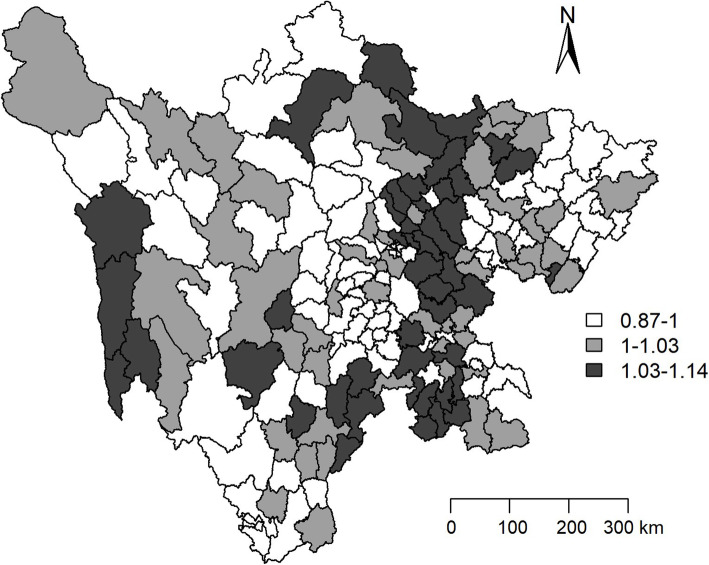
Maps of the distribution of the estimated county level *RR*s after adjusting the social-economic variables in Sichuan province. The deeper the color and the higher the *RR*s of posteriori distribution. In comparison with the average decrease tendency of TB in Sichuan province from 2006 to 2015, the areas with dark grey had the faster decreasing of the TB notification rate

The data in the Figs. 4 and 5 indicate the estimated county level posterior mean smoothed and *RR*s for temporal effect in Sichuan province. The dark greys were mainly distributed in the east-central regions, which meant that those counties had higher decrease tendency of TB CNR compared with the average decrease tendency of total 181 counties in Sichuan province from 2006 to 2015.

## Discussion

The hierarchical Bayesian spatio-temporal model provides a robust way to examine the association between TB CNR and its socio-economic risk factors. In this analysis, we found that the TB CNR was associated with a quadratic trend of time in Sichuan province from 2006 to 2015, and it showed that the heavy TB burden in Sichuan province mainly attributed to ageing and less education year after adjusted the spatial effect, temporal effect, and spatio-temporal interactions. The higher education year and the lower proportion of age group were related to the lower the TB CNR.

The TB CNR decreased by 27.25% from 2006 to 2015 in Sichuan province, with annual reported notification incidence 77 per10^5^, which was lower than that in China (81.46 per10^5^) [29]. The TB CNR increased from 2006 to 2007 but decreased since 2007. The Directly Observed Treatment Short-Course strategy was expanded nation-wide to 100% coverage in 2005 [30], thus the TB case detection was increased at the beginning and then decreased. Sichuan province has achieved great progress on TB control. The number of TB medical care workers decreased from 1377 to 1095 persons from 2010 to 2017, and also the funds for TB prevention and control decreased from 111 million to 49.32 million from 2010 to 2017 in Sichuan province. However, the incidences of HIV/TB coinfection, chronic disease with TB and multi-drug resistant TB have increased in recent years [31, 32]. Thus, the current TB control situation remains seriously.

The TB CNR was spatial heterogeneously distributed, and we found positive spatial autocorrelation of TB CNR from 2006 to 2015 in Sichuan province according to the global Moran’s *I*, with the trend of increasing global Moran values over time, which indicated that neighbor counties were more likely to interact with each other. This might be due to the convenient transportation and population mobility among those counties. The global Moran’s *I* index also indicated that spatial dependence in the residuals was not neglected when exploring the socio-economic risk factors for TB CNR. In this study, we found the spatio-temporal interaction effect parameter was statistically significant, thus, spatio-temporal analysis in this study is superior to the traditional analysis [33–35].

The TB cases were closely related to the socio-economic status of the population, Janssens [36] had indicated that we must fight poverty to stop TB. People with lower socio-economic status are more likely to be exposed to active TB patients, living in crowded places with poor ventilation, and also with lower awareness of unhealthy behaviors (such as smoking and alcohol drinking). However, in the univariate analysis, we found a positive association between PGDP and TB CNR, which is contradictory to other studies [10, 37]. When we used a spatial model to explore the risk factors, and a negative relationship between PGDP and TB CNR was found. Furthermore, a higher positive correlation between PGDP and time was observed, with a Pearson coefficient of 0.61. Thus, we indicated that the collinear situation was the main reason to explain this inconsistent result. At last, considering collinear and accuracy, in the multivariate analysis, the PGDP variable was not put into the model.

We found the proportion of age group over 60 years old was a risk factor for the TB notification rate in Sichuan province. Because of underlying diseases, malnutrition and biological changes with aging, the elder people have a lower cellular immune response to *M. tb* [38]. With age increasing, the Vitamin D made by skin reduced [39], which plays an important role in the calcium metabolism of hormones [40]. Lacking serum Vitamin D has been suggested to be related to increased risk of cancer, diabetes and TB [41]. In the univariate analysis, there was a negative relationship between sex ratio (male to female) and TB CNR, which indicated that the higher proportional of the male was associated with the lower risk of TB CNR. Guo [11] had also addressed that the female was the risk factor for TB in China from 2005 to 2013. The reason why the female was a risk for the TB CNR we had previously illustrated [32]. Although the sex ratio was not included in the last model, the potential effect of the female should be paid more attention to. We found there was a negative association between education year and TB CNR. The education year was the most important risk factor in either univariate or multivariate models. TB is an infectious disease and closely related to the body’s immunity, and which is affected by the quality of life and living habits. The population with higher education always has more potentially higher wealth status, hygiene knowledge, and healthy behaviors, which may prevent them from infectious diseases. Thus, to reduce the TB CNR in Sichuan province, improving the education level in population is the most important strategy for the government.

This study also had some limitations. Firstly, reported TB cases were from TB surveillance system, which may be affected by the missing report and under-diagnosis in some poor counties, especially in the Aba prefecture, Ganzi prefecture and Lianshan prefecture, and resulted in the underestimate of TB CNR. Secondly, some important social-economic variables such as funding, HIV epidemic, diagnosis capacity, Bacillus Calmette-Guerin vaccination, and accessibility of TB health services such as cure rate or relapse of sputum smear-positive TB cases were not be collected, which may raise some bias. Thirdly, five types of social-economic variables mentioned above may be not enough to summarize all the social-economic types and some variables may have more than one characteristic, which may result in misclassification. Fourthly, as for an ecological study, ecological fallacy may be unavoidable. However, the evidence from the ecological study can provide some clues for the government to control and prevent the TB epidemic.

Despite those limitations, the spatial dependence of the phenomenon and temporal autocorrelations and spatio-temporal interactions model which has been used in this study to avoiding biases in the estimates.

## Conclusions

There were spatial clusters of TB CNR in Sichuan province from 2006 to 2015. This research suggests that the proportion of age group and people with low socioeconomic status including poor education are associated with increasing TB CNR. It is more important to pay more attention to the elderly population and improve socioeconomic status including promoting education level in Sichuan province to reduce the TB burden.

## Supplementary information

**Additional file 1.** The weights plot of Queen in 181 counties of Sichuan province. The common boundaries in 181 counties of Sichuan province can be clearly distinguished, so the queen weights were used in this manuscript.

**Additional file 2.** The DICs of the five models under different priors’ distribution without social-economic variables. The minimum DIC was 19,379.01 and the model V was the best model with the prior information with the Gamma distribution (0.1,0.1) and the default prior information with uniform distribution (0,1000).

**Additional file 3.** The posteriori distributions of parameters in the parametric spatio-temporal interactions model without socio-economic covariates with the prior information with the Gamma distribution (0.1,0.1) and the default prior information with uniform distribution (0,1000) in 181 counties of Sichuan province, 2006 to 2015.

**Additional file 4.** The residuals plot of the fitted Spatio-temporal model after adjusting for education year and proportion of age in Sichuan province.

## Data Availability

The dataset used and analyzed during the current study is available from the corresponding author on reasonable request.

## Abbreviations

TB: Tuberculosis
HIV: Human immunodeficiency virus
WHO: World health organization
CNR: Case notification rate
*M.tb*: *Mycobacterium tuberculosis*
PGDP: Per gross domestic product
CNY: Chinese Yuan
PD: Population density
NBMI: Number of beds in medical institutions
NMW: Medical personnel per 1000 persons
DIC: Deviance information criterion
SD: Standard deviation
CAR: Conditional auto-regression
AR1: Autoregressive model of order 1
RR: Relative risk
CI: Credible interval
INLA: Integrated nested laplace approximation
P_25_: 25% quartile
P_50_: Median
P_75_: 75% quartile
