# Supplementary material for: Spatio-temporal analysis of socio-economic characteristics for pulmonary tuberculosis in Sichuan province of China, 2006–2015
Source: BMC Infect Dis. 2020 Jun 22;20:433. doi: 10.1186/s12879-020-05150-z (PMC7310234; doi:10.1186/s12879-020-05150-z)
Supplement: Supplementary file 1 — Additional file 1. The weights plot of Queen in 181 counties of Sichuan province. The common boundaries in 181 counties of Sichuan province can be clearly distinguished, so the queen weights were used in this manuscript. [file 12879_2020_5150_MOESM1_ESM.pptx]

## Slide 1
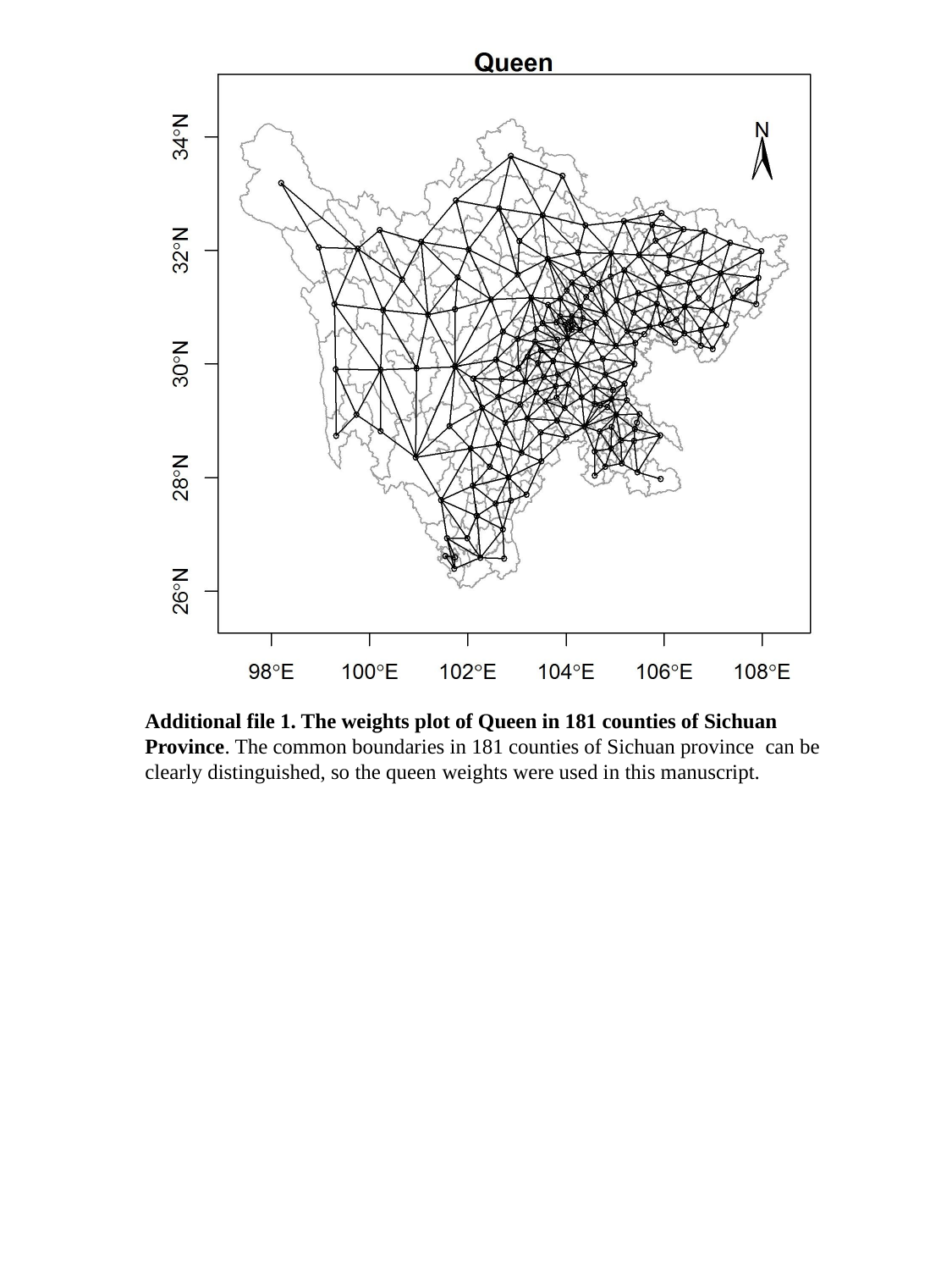

Additional file 1. The weights plot of Queen in 181 counties of Sichuan Province. The common boundaries in 181 counties of Sichuan province can be clearly distinguished, so the queen weights were used in this manuscript.
