# Supplementary material for: Spatio-temporal analysis of socio-economic characteristics for pulmonary tuberculosis in Sichuan province of China, 2006–2015
Source: BMC Infect Dis. 2020 Jun 22;20:433. doi: 10.1186/s12879-020-05150-z (PMC7310234; doi:10.1186/s12879-020-05150-z)
Supplement: Supplementary file 4 — Additional file 4. The residuals plot of the fitted Spatio-temporal model after adjusting for education year and proportion of age in Sichuan province. [file 12879_2020_5150_MOESM4_ESM.pptx]

## Slide 1
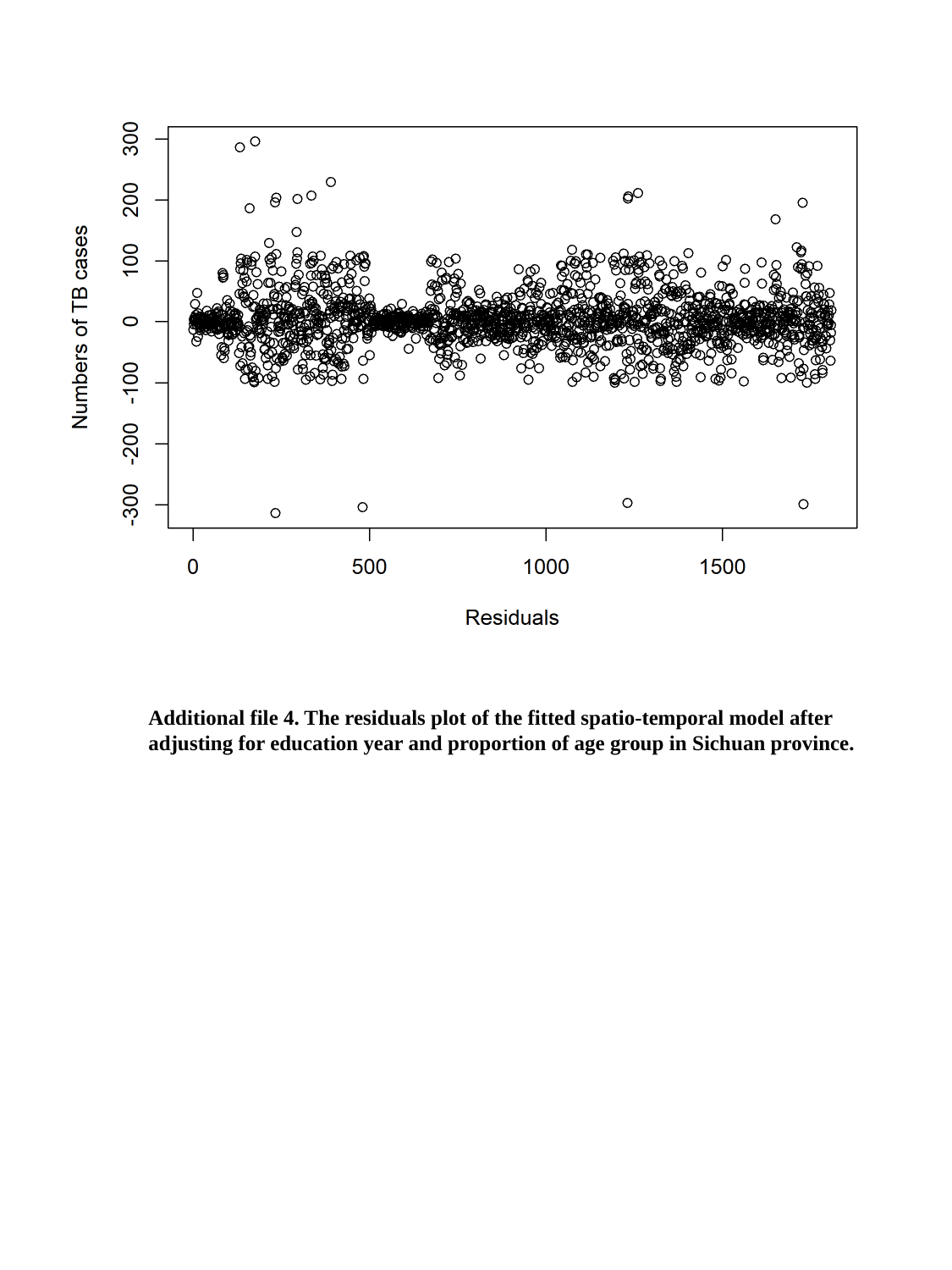

Additional file 4. The residuals plot of the fitted spatio-temporal model after adjusting for education year and proportion of age group in Sichuan province.
